# Observed changes in clinical proxy measures of airway protection after cervical epidural spinal cord stimulation in neurological disorders: a preliminary retrospective case series

**DOI:** 10.3389/fneur.2026.1875581

**Published:** 2026-07-23

**Authors:** Guofeng Zhang, Zemin Li, Xiaoli Yao, Minghui Ding, Xuesong Liu

**Affiliations:** 1 Department of Neurosurgery, The First Affiliated Hospital, Sun Yat-sen University, Guangzhou, Guangdong, China; 2 Department of Neurology, The First Affiliated Hospital, Sun Yat-sen University, Guangzhou, Guangdong, China; 3 Department of Rehabilitation Medicine, The First Affiliated Hospital, Sun Yat-sen University, Guangzhou, Guangdong, China

**Keywords:** airway protection, cough reflex, dysphagia, neuromodulation, spinal cord stimulation

## Abstract

**Objectives:**

Although spinal cord stimulation (SCS) has been investigated for cough and respiratory motor output after neurological injury, its relationship with clinical proxy measures of airway protection remains unclear. We explored observed changes in aspects of airway protection after cervical SCS in patients with neurological disorders.

**Materials and methods:**

We retrospectively analyzed six patients who underwent cervical SCS for spinal cord injury, amyotrophic lateral sclerosis, or disorders of consciousness. Clinical proxy measures included the Semi-quantitative Cough Strength Score (SCSS), Functional Oral Intake Scale (FOIS), Water Swallow Test (WST), and Chinese Swallowing Quality of Life Questionnaire (cSWAL-QoL). SCSS was available for all six patients, whereas FOIS, WST, and cSWAL-QoL were limited to the four conscious patients. SCSS, FOIS, and WST were descriptively compared across preoperative, stimulation ON, and stimulation OFF conditions when clinically permissible. cSWAL-QoL was compared between preoperative and postoperative follow-up.

**Results:**

During active stimulation, SCSS scores were higher in all six patients, with the median score changing from 3.0 preoperatively to 4.0 during stimulation ON (nominal *p* = 0.031). SCSS was also higher during stimulation ON than OFF (4.0 vs. 3.0; nominal *p* = 0.031). Among the four conscious patients, FOIS changed from 4.0 to 5.0 and WST changed from 3.5 to 2.5 during stimulation ON, while cSWAL-QoL changed from 91.0 to 151.5 postoperatively. No stimulation-related adverse events occurred.

**Conclusion:**

Clinical proxy measures related to airway protection showed exploratory state-associated changes during cervical epidural SCS, particularly in cough-strength scores. These preliminary findings do not establish efficacy or mechanism.

## Introduction

Swallowing dysfunction and impaired airway protective reflexes are common and clinically significant complications in patients with neurological disorders, including spinal cord injury (SCI), amyotrophic lateral sclerosis (ALS), and disorders of consciousness (DOC). These impairments compromise nutritional intake and quality of life and increase the risk of aspiration pneumonia, a major contributor to morbidity and mortality in neurologically vulnerable populations.

Airway protection relies on the tightly coordinated interaction between coughing, swallowing, and respiration, forming an integrated brainstem-mediated defense system. Cough clears airway secretions and aspirated material, swallowing prevents bolus entry into the airway, and respiratory timing determines whether these behaviors occur safely and efficiently. Disruption of any component can therefore impair the entire protective network. In clinical observational studies, however, airway protection is often inferred from proxy measures such as cough strength, oral intake, swallowing safety, and aspiration-related clinical observations rather than directly measured as a single physiological construct.

Epidural spinal cord stimulation (SCS) has emerged as a neuromodulation strategy for restoring motor and autonomic functions after neurological injury. In particular, prior clinical and experimental studies have shown that SCS can augment cough function by activating expiratory musculature, increasing intrathoracic pressure, and improving airway clearance in patients with spinal cord injury (1–9). These findings raise the possibility that SCS may influence broader airway protective functions beyond isolated respiratory motor output.

Despite this rationale, the effect of cervical SCS on swallowing function and cough effectiveness remains largely unexplored. Most prior work has focused on respiratory mechanics, motor recovery, or arousal-related outcomes, whereas the integrated modulation of brainstem-mediated airway protection has received limited attention (10–12). This gap is important because coughing, swallowing, and respiration share partially overlapping neural substrates and are highly interdependent during airway defense.

Recent experimental studies further suggest that cervical epidural stimulation can influence respiratory networks and spinal-respiratory plasticity (13–18). In parallel, clinical studies have documented dysphagia and impaired respiratory-swallow coordination after cervical SCI (19–23) or stroke (24). However, direct clinical evidence linking cervical SCS to swallowing-related changes and clinical proxy measures of airway protection remains scarce.

In this study, we retrospectively analyzed a cohort of six patients who underwent cervical epidural SCS for indications unrelated to dysphagia treatment, including SCI, ALS, and DOC. Four patients were conscious and two were comatose. The inclusion of patients with DOC allowed exploratory assessment of airway protective responses independent of voluntary participation, but these patients could not undergo formal swallowing assessments. Observed changes in cough reflex, swallowing-related performance, and swallowing-related quality of life were noted after SCS implantation. We explored the hypothesis that cervical epidural SCS may influence clinical proxy measures related to an integrated airway protection network by interacting with spinal-brainstem circuits involved in cough, swallowing, and respiratory coordination. This study aims to provide preliminary, hypothesis-generating clinical observations and to describe the pattern of state-associated score changes using multiple complementary clinical scales.

## Materials and methods

### Study design and patient selection

This study was a retrospective case series conducted at a single center from April 2025 to March 2026. Patients were eligible for inclusion if they underwent cervical epidural spinal cord stimulation (SCS) and had available clinical data regarding airway protective reflexes and swallowing-related function before and after implantation. Six consecutive patients were included in the final analysis. Because the study was retrospective and exploratory, no formal sample size calculation was performed; the sample size was determined by the number of eligible patients treated during the study period. The cohort comprised patients with cervical spinal cord injury, amyotrophic lateral sclerosis, and disorders of consciousness. This diagnostic heterogeneity was retained to reflect real-world clinical practice but was considered an important limitation for interpretation. In all cases, cervical epidural SCS had been implanted for clinical indications unrelated to the primary treatment of dysphagia, including pain and increased muscle tone, decline in muscle strength, and arousal facilitation. Baseline demographic and clinical variables were extracted from the medical record, including age, sex, primary diagnosis, etiology, time from onset to SCS, neurological status, consciousness state, tracheostomy status, ventilatory support, feeding route, cough reflex, expectoration ability, swallowing/feeding status, suction frequency, aspiration risk or pulmonary infection history, airway management status, and follow-up duration. This study was approved by the Institutional Review Board of our institution. Since this was a retrospective study, the requirement for written informed consent was waived.

### Surgical procedure and stimulation protocol

All patients underwent epidural implantation of cervical spinal cord stimulation leads and Implantable Pulse Generator (Medtronic plc or PINS Medical Co., Ltd.). Electrode target levels ranged from C2–4 to C6–T1, and two patients received combined cervical and thoracolumbar lead placement. Stimulation mapping was performed in all patients. Stimulation settings were individualized according to disease type, electrode location, and therapeutic indication. Programmed stimulation parameters included amplitude, frequency, and pulse width, with patient-specific daily exposure schedules, including day-night amplitude switching, alternating low- and high-frequency stimulation, continuous stimulation, or intermittent daytime cycling. Concomitant rehabilitation with or without airway care was administered according to the clinical condition of each patient.

### Clinical assessment

Airway protection and swallowing-related function were evaluated using clinical proxy measures rather than comprehensive physiological testing. Specifically, airway protection was inferred from routinely documented clinical measures of cough effectiveness, secretion clearance, oral intake, swallowing safety, and swallowing-related quality of life. The Semi-quantitative Cough Strength Score (SCSS) was used to assess cough effectiveness and airway clearance capacity in all six patients, with higher scores indicating stronger and more effective cough capable of clearing airway secretions. Impaired SCSS is associated with an increased risk of secretion retention and aspiration. For conscious patients, SCSS was scored according to the documented cough response during routine airway clearance assessment, including voluntary cough when possible and reflexive cough or airway response during clinical stimulation or suctioning when applicable. For comatose patients, SCSS was assessed only according to airway response to suctioning or airway stimulation and was not interpreted as a swallowing assessment. The Functional Oral Intake Scale (FOIS) was used to assess the level of oral intake, ranging from complete tube dependence to full oral intake without restriction. Higher scores indicate better oral feeding ability. The Water Swallow Test (WST) was used to assess swallowing safety. Lower scores indicate safer swallowing performance and lower aspiration risk. The Chinese version of the Swallowing Quality of Life Questionnaire (cSWAL-QoL) was used to evaluate patient-reported swallowing-related quality of life (25). The scores range from 0 to 220, and higher scores indicate better swallowing-related quality of life. FOIS, WST, and cSWAL-QoL were assessed in the four conscious patients who were able to undergo functional swallowing evaluation and complete patient-reported measures. All outcome assessments were performed according to the clinical follow-up protocol by the same neurosurgical physician, the same nursing team responsible for airway care, and the same neurorehabilitation team throughout the study period. The scoring criteria and interpretation of SCSS and swallowing-related assessments were standardized through consensus between neurosurgeons and rehabilitation physicians before data extraction and analysis.

### Assessment time points

Quantitative assessments were collected under different stimulation conditions according to the available dataset. SCSS, FOIS, and WST scores were recorded at three time points: preoperative baseline, defined as the baseline assessment before SCS implantation; stimulation ON, defined as assessment during the clinically programmed active stimulation state after implantation when clinical changes were observed; and stimulation OFF, defined as assessment after implantation with stimulation temporarily turned off for one day when clinically permissible. The OFF condition was not randomized, blinded, or based on a standardized experimental washout protocol. Because stimulation schedules and postoperative assessment windows varied across patients, the ON and OFF comparisons should be interpreted as within-subject exploratory observations rather than standardized experimental washout tests. For cSWAL-QoL, scores were available at two time points only: preoperative and postoperative assessment after clinically observed functional change. In addition to scale-based assessments, follow-up records were reviewed for stimulation protocols, assessment intervals, cough outcome, secretion clearance, swallowing/feeding outcome, and adverse events.

### Outcomes

The primary outcome of this study was to describe observed changes in clinical proxy measures related to airway protective and swallowing-related function after cervical epidural SCS, as measured by SCSS, FOIS, WST, and cSWAL-QoL. Secondary descriptive outcomes included changes in secretion clearance, suction requirement, oral intake status, and the presence or absence of stimulation-related adverse events. The analysis was intended to describe within-subject observations rather than to establish efficacy.

### Data handling and statistical analysis

Continuous or ordinal clinical scale data were summarized as median and interquartile range (IQR) because of the small sample size and non-normal distribution assumptions. Categorical variables were summarized descriptively using counts and proportions where appropriate. SCSS, FOIS, and WST were treated as paired ordinal outcome measures across repeated within-subject conditions. cSWAL-QoL was treated as a paired continuous summary score for preoperative and postoperative comparison. Statistical analyses were performed using paired non-parametric methods because of the small sample size and the ordinal nature of several outcome measures. For SCSS, FOIS, and WST, paired comparisons were conducted between preoperative and stimulation ON conditions, stimulation ON and stimulation OFF conditions, and preoperative and stimulation OFF conditions using the Wilcoxon signed-rank test. For cSWAL-QoL, preoperative and postoperative scores were compared using the Wilcoxon signed-rank test. Descriptive results are presented as median (IQR), together with individual patient trajectories where applicable. Given the extremely small sample size, all statistical tests were exploratory, and *p* values were retained only for transparency and interpreted as nominal and descriptive rather than as definitive evidence of treatment effect. No adjustment for multiple comparisons was performed, and no definitive statistical inference was drawn from these tests.

## Results

### Patient characteristics

Six patients who underwent cervical epidural spinal cord stimulation were included in this case series. Baseline characteristics are summarized in Table 1. The cohort comprised two patients with traumatic cervical spinal cord injury, two with amyotrophic lateral sclerosis, one with hypoxic–ischemic encephalopathy secondary to postoperative cervical hematoma after thyroid surgery, and one with intracerebral hemorrhage. Age ranged from 52 to 68 years, and five patients were male. Time from disease onset to SCS implantation ranged from 4 to 48 months. Four patients were awake at baseline, whereas two were comatose. Among the spinal cord injury cases, the neurological impairment was classified as American Spinal Injury Association Impairment Scale (AIS) grade A in one patient and grade B in the other. Baseline neurological deficits varied according to etiology, ranging from complete quadriplegia and severe sensory impairment in traumatic cervical spinal cord injury, to progressive weakness, diffuse muscle atrophy, dysarthria, and dysphagia in amyotrophic lateral sclerosis, and markedly impaired responsiveness with a Coma Recovery Scale-Revised (CRS-R) score of 7 in the two comatose patients.

**Table 1:** Baseline demographic, neurological, airway, and swallowing characteristics of the study cohort.

Patient	Demographics and disease characteristics					Baseline neurological status		
	Age (yrs)	Sex	Primary diagnosis	Etiology	Time from onset to SCS (months)	Baseline neurological status	Consciousness status	Neurological level (AIS grade)
Patient 1	58	M	Cervical spinal cord injury	Fall	4	Hypoesthesia below C6, anesthesia below T4, increased tone in both upper extremities, and 0/5 strength in all extremities	Awake	A
Patient 2	61	M	Cervical spinal cord injury	Motor vehicle trauma	48	2/5 strength in all extremities, increased tone, Modified Ashworth Scale grade 3	Awake	B
Patient 3	68	M	ALS	ALS	19	Upper-extremity strength 3/5, lower-extremity strength 5-/5, diffuse muscle atrophy, dysarthria, and dysphagia	Awake	NA
Patient 4	63	M	ALS	ALS	18	Upper-extremity strength 2/5, lower-extremity strength 3/5, increased lower-extremity tone, diffuse muscle atrophy, dysarthria, and dysphagia	Awake	NA
Patient 5	56	F	Hypoxic ischemic encephalopathy	Hypoxia secondary to postoperative cervical hematoma after thyroid surgery	34	Motor response grade 2 in all extremities with increased tone; CRS-R score of 7	Coma	NA
Patient 6	52	M	Intracerebral hemorrhage	Intracerebral hemorrhage	9	Motor response grade 2 in all extremities with increased tone; CRS-R score of 7	Coma	NA

Patient	Baseline airway and swallowing status						Airway risk and follow-up			
	Tracheostomy	Ventilatory support	Baseline feeding route	Baseline cough reflex	Baseline expectoration ability	Baseline swallowing/ feeding status	Suction frequency (times/day)	Aspiration risk or pulmonary infection history	Baseline airway management	Follow-up duration (months)
Patient 1	Yes	No	Nasogastric tube	Weak	Partially effective	Severe dysphagia with absent safe oral intake	8–10	Yes	Intermittent suction dependent	12
Patient 2	No	No	Oral feeding	Weak	Partially effective	Full oral intake with difficulty	0	Yes	Spontaneous airway	7
Patient 3	No	No	Oral feeding	Weak	Partially effective	Able to take soft diet	0	Yes	Spontaneous airway	6
Patient 4	No	No	Oral feeding	Weak	Partially effective	Able to take soft diet	0	Yes	Spontaneous airway	1
Patient 5	Yes	No	Nasogastric tube	Weak	Ineffective	Absent swallowing response	10–15	Yes	Intermittent suction dependent	10
Patient 6	Yes	No	Nasogastric tube	Absent	Ineffective	Absent swallowing response	8–10	Yes	Intermittent suction dependent	9

AIS, American Spinal Injury Association Impairment Scale; ALS, amyotrophic lateral sclerosis; CRS-R, Coma Recovery Scale-Revised; SCS, spinal cord stimulation.

Baseline airway and swallowing compromise was present across the cohort, although severity varied. Three patients had tracheostomies, three required nasogastric tube feeding, and none required ventilatory support. Baseline cough reflex was weak in five patients and absent in one. Expectoration was partially effective in four patients and ineffective in two. Swallowing status ranged from full oral intake with difficulty or soft-diet intake in the awake patients to absent swallowing response in the two comatose patients. All six patients had a documented history of aspiration risk or pulmonary infection. Suction frequency ranged from 8 to 15 times per day for patients with tracheostomies, and baseline airway management was spontaneous in three patients and intermittent suction-dependent in three. Follow-up duration ranged from 1 to 12 months.

### Stimulation characteristics, exposure protocols, and clinical outcomes

All six patients underwent cervical epidural spinal cord stimulation with stimulation mapping performed in every case (Table 2). Electrode target levels ranged from C2-4 to C6-T1. Two patients underwent combined cervical and thoracolumbar implantation (Patient 2: C4-7 and T10-12; Patient 4: C3-5 and T12-L1), whereas the remaining four patients received cervical leads only. The indication for cervical epidural stimulation was pain and increased muscle tone in Patients 1 and 2, decline in muscle strength in Patients 3 and 4, and arousal facilitation in Patients 5 and 6.

**Table 2:** Stimulation protocol, exposure schedule, and clinical observations after cervical epidural spinal cord stimulation.

Patient	Assessment schedule				Stimulation protocol		
	Lead configuration	Indication	Mapping	Target levels	Active settings	Daily exposure	Concomitant therapy
Patient 1	2*8 surgical lead in C6-T1	Pain and increased muscle tone	Yes	C6	0.65-1.20 V, 40 Hz, 200 μs	Session 1 (8 AM-9 PM): amplitude 1.20 V; Session 2 (9 PM-8 AM next day): amplitude 0.65 V.	Rehabilitation airway care
Patient 2	2*8 surgical lead in C4-7, 5-6-5 surgical lead in T10-12	Pain and increased muscle tone	Yes	C4-5, T11-12	Cervical and lumbar: 0.7 0.85 V, 10 Hz, 210 μs	Session 1 (8 AM-9 PM): Amplitude 0.85 V; Session 2 (9 PM-8 AM next day): amplitude 0.7 V.	Rehabilitation
Patient 3	2*8 surgical lead in C4-7	Decline in muscle strength	Yes	C4	0.9-2.1 mA, 5 Hz & 60 Hz, 200 μs	Session 1 (9AM-11AM & 2PM-4PM): frequency 5 Hz; Session 2 (rest of the day): frequency 60 Hz	Rehabilitation
Patient 4	2*8 surgical lead in C3-5, 5-6-5 surgical lead in T12-L1	Decline in muscle strength	Yes	C3-4, T12	Cervical: 1.65 mA, 50 Hz, 320 μs; Lumbar: 0.3 mA, 30 Hz, 300 μs	24 hours/day	Rehabilitation
Patient 5	2*8 surgical lead in C2-4	Arousal facilitation	Yes	C2	0.65 V, 70 Hz, 210 μs	Session 1 (8 AM-8 PM): stimulation ON and OFF at 15-minute intervals; Session 2 (rest of the day): stimulation OFF	Airway care
Patient 6	2*8 surgical lead in C2-4	Arousal facilitation	Yes	C2	1.80 V, 70 Hz, 190 μs	Session 1 (8 AM-8 PM): stimulation ON and OFF at 15-minute intervals; Session 2 (rest of the day): stimulation OFF	Airway care

Patient	Assessment schedule		Clinical observations			
	Assessment intervals	Primary endpoint timepoint	Cough observation	Secretion clearance	Swallowing/feeding	Adverse events
Patient 1	Daily during the first week; weekly within month 1; monthly within the first 3 months; every 6 months thereafter; anytime in emergency.	12 weeks	Higher cough effectiveness observed	Suction Frequency decreased (5–6 times/day); more efficient clearance observed	Oral intake observed after prior tube feeding	No
Patient 2	Daily during the first week; weekly within month 1; monthly within the first 3 months; every 6 months thereafter; anytime in emergency.	8 weeks	Higher cough effectiveness observed	More efficient clearance observed	Higher oral intake level observed	No
Patient 3	Daily during the first week; weekly within month 1; monthly within the first 3 months; every 6 months thereafter; anytime in emergency.	1 day	Higher cough effectiveness observed	More efficient clearance observed	Higher oral intake level observed	No
Patient 4	Daily during the first week; weekly within month 1; monthly within the first 3 months; every 6 months thereafter; anytime in emergency.	1 week	Higher cough effectiveness observed	More efficient clearance observed	Higher oral intake level observed	No
Patient 5	Daily during the first week; monthly within the first 3 months; every 6 months thereafter; anytime in emergency.	4 weeks	Stronger cough reflex observed	Suction frequency decreased (5–6 times/day); more efficient clearance observed	Tube feeding unchanged	No
Patient 6	Daily during the first week; daily during the first week; monthly within the first 3 months; every 6 months thereafter; anytime in emergency.	1 day	Stronger cough reflex observed	Suction frequency decreased (4–5 times/day); more efficient clearance observed	Tube feeding unchanged	No

Stimulation settings were individualized according to level and indication. Across the cohort, programmed amplitudes ranged from 0.65 to 1.80 V or 0.3 to 2.1 mA, stimulation frequencies ranged from 5 to 70 Hz, and pulse width ranged from 190 to 320 μs. Exposure schedules varied across patients, including day-night amplitude switching (Patients 1 and 2), alternating low-frequency and high-frequency stimulation (Patient 3), continuous 24 h/day stimulation (Patient 4), and daytime intermittent ON/OFF cycling with stimulation OFF during the remainder of the day (Patients 5 and 6). Concomitant treatment included rehabilitation, airway care, or both.

Follow-up assessment was performed daily during the first postoperative week in all patients, followed by protocol-specific weekly, monthly, and longer-term surveillance. Primary endpoint capture timepoints ranged from day 1 to week 12. Observed increases in cough reflex or cough effectiveness were recorded in all six patients. More efficient secretion clearance was documented in all six patients, with reduced suction frequency in Patients 1, 5 and 6. Swallowing or feeding records showed observed favorable changes in the four conscious patients, including transition from tube feeding to oral intake in Patient 1 and higher oral intake levels in Patients 2–4, whereas tube feeding remained unchanged in Patients 5 and 6. No perioperative adverse events or stimulation-related complications were observed during follow-up.

### Descriptive analysis of clinical proxy measures related to airway protection and swallowing

**Figure 1:**
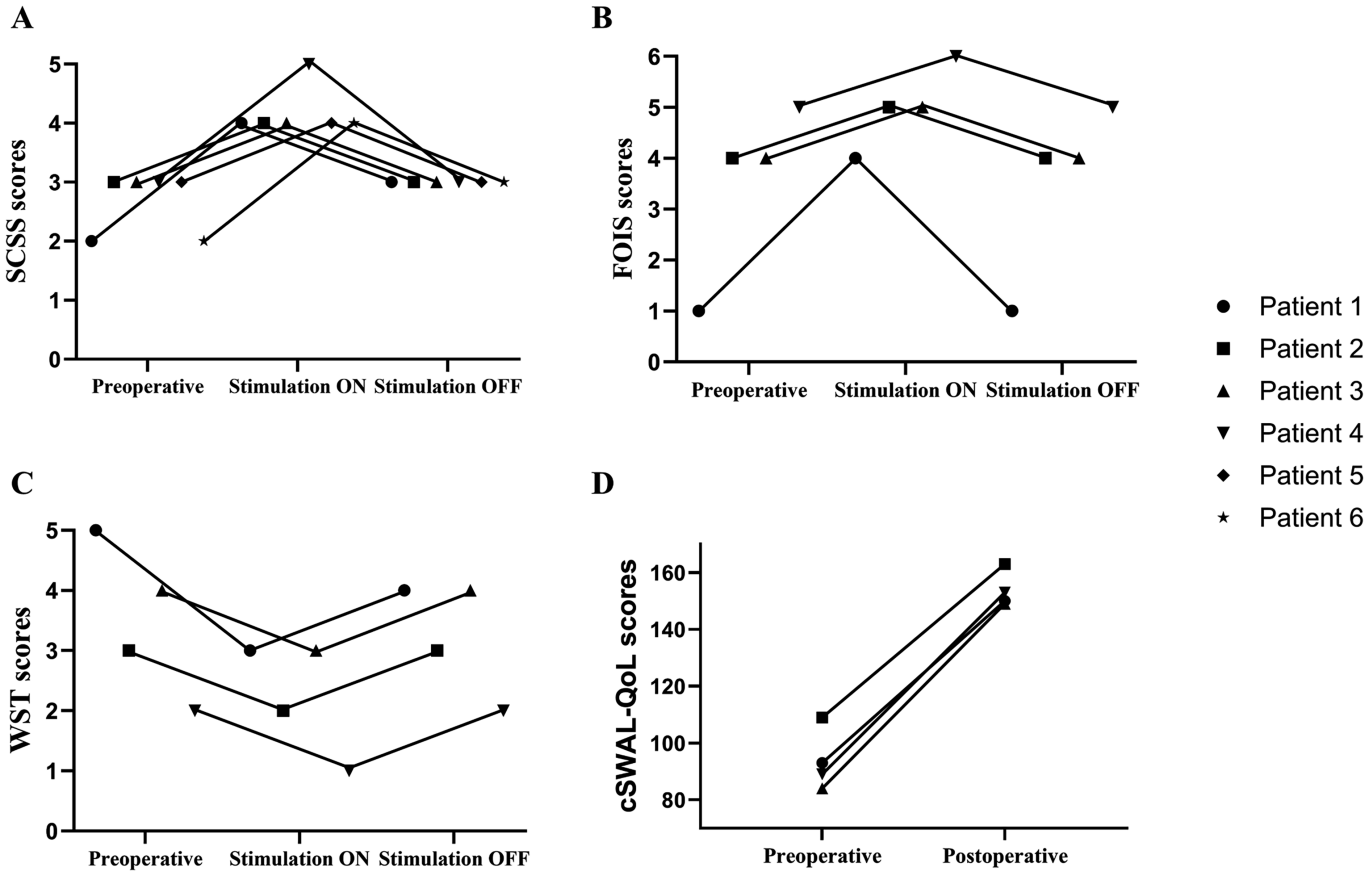
Observed changes in clinical proxy measures related to cough strength, swallowing function, and swallowing-related quality of life after cervical epidural spinal cord stimulation. **(A)** Semi-quantitative Cough Strength Score (SCSS) in six patients across preoperative, stimulation ON, and stimulation OFF conditions. Higher scores indicate stronger cough and better airway clearance. **(B)** Functional Oral Intake Scale (FOIS) in four conscious patients across preoperative, stimulation ON, and stimulation OFF conditions. Higher scores indicate better oral intake ability. **(C)** Water Swallow Test (WST) scores in four conscious patients across preoperative, stimulation ON, and stimulation OFF conditions. Lower scores indicate safer swallowing and lower aspiration risk. **(D)** The Chinese version of the Swallowing Quality of Life Questionnaire (cSWAL-QoL) scores in four conscious patients across preoperative and postoperative conditions. Higher scores indicate better swallowing-related quality of life. Each line represents an individual patient trajectory. Panels **(A–C)** show exploratory within-subject observations across non-standardized stimulation states, while panel **(D)** shows postoperative changes in patient-reported swallowing-related quality of life. cSWAL-QoL, Chinese version of the Swallowing Quality of Life Questionnaire; FOIS, Functional Oral Intake Scale; SCSS, Semi-quantitative Cough Strength Score; WST, Water Swallow Test.

To describe clinical proxy measures potentially related to airway protection, we summarized cough-strength, oral-intake, swallowing-safety, and patient-reported swallowing-related quality-of-life scores. These measures should be interpreted as clinical proxies rather than comprehensive physiological assessment of airway protection. SCSS data were obtained in all six patients under preoperative, stimulation ON, and stimulation OFF conditions. The median SCSS changed from 3.0 (IQR 2.25–3.0) preoperatively to 4.0 (IQR 4.0–4.0) during stimulation ON, and then to 3.0 (IQR 3.0–3.0) during stimulation OFF. The Wilcoxon signed-rank test yielded a nominal *p* value of 0.031 for the comparison between preoperative and stimulation ON conditions and 0.031 for the comparison between stimulation ON and stimulation OFF conditions, whereas the difference between preoperative and OFF conditions yielded nominal *p* = 0.157. Because only six patients were included, these *p* values should be interpreted as descriptive and exploratory. At the individual level, all six patients showed improved SCSS during stimulation ON; Patients 1 and 6 remained numerically above baseline in the OFF condition, whereas Patients 2–5 returned to baseline values (Figure 1A). This pattern is consistent with state-associated variation in cough strength within this cohort, but it does not establish stimulation-dependent efficacy.

FOIS was assessed in the four conscious patients. The median FOIS score changed from 4.0 (IQR 3.25–4.25) preoperatively to 5.0 (IQR 4.75–5.25) during stimulation ON, and returned to 4.0 (IQR 3.25–4.25) during stimulation OFF. These differences did not reach statistical significance (preoperative vs. ON, nominal *p* = 0.125; ON vs. OFF, nominal *p* = 0.125). At the individual level, all four conscious patients had higher FOIS scores during stimulation ON (Figure 1B). Patient 1 changed from tube-dependent feeding (FOIS 1) to a score of 4 during stimulation ON, while Patients 2 and 3 changed from 4 to 5 and Patient 4 changed from 5 to 6. In all four patients, the OFF value returned to the preoperative level. These observations are limited to conscious patients and should not be generalized to the two patients with DOC.

WST was also available in the four conscious patients. Because lower WST scores indicate better swallowing safety, the observed score changes were directionally favorable during stimulation ON. The median WST score changed from 3.5 (IQR 2.75–4.25) preoperatively to 2.5 (IQR 1.75–3.0) during stimulation ON, and then to 3.5 (IQR 2.75–4.0) during stimulation OFF. These differences did not reach statistical significance (preoperative vs. ON, nominal *p* = 0.125; ON vs. OFF, nominal *p* = 0.125; preoperative vs. OFF, nominal *p* = 0.317). At the individual level, all four conscious patients showed lower WST scores during stimulation ON than at baseline, while the OFF value returned to baseline in Patients 2–4 and remained slightly lower in Patient 1 (Figure 1C).

The cSWAL-QoL was completed in the four conscious patients and was recorded under preoperative and postoperative conditions. The median cSWAL-QoL score changed from 91.0 (IQR 87.75–97.0) preoperatively to 151.5 (IQR 149.75–155.5) postoperatively. This difference did not reach statistical significance in this small cohort (nominal *p* = 0.125). Individual scores changed from 93 to 150, 109 to 163, 84 to 149, and 89 to 153, respectively (Figure 1D). These patient-reported observations were not available for patients with DOC.

Taken together, these data show directionally consistent observed changes in clinical proxy measures related to airway protection and swallowing during stimulation ON, with the clearest signal observed in SCSS. FOIS and WST values generally returned toward baseline during stimulation OFF, suggesting a possible stimulation-state pattern in this dataset. By comparison, cSWAL-QoL showed postoperative increases in all four conscious patients. Because the cohort was small and heterogeneous, the OFF condition was not a standardized washout protocol, and objective physiological measures were not systematically available, these observations should be interpreted as preliminary and hypothesis-generating rather than definitive evidence of efficacy.

## Discussion

### Principal findings

In this small, heterogeneous retrospective case series, cervical epidural SCS was associated with observed changes in clinical proxy measures related to cough strength and swallowing-related function. Airway protection was inferred from SCSS, FOIS, WST, and cSWAL-QoL rather than directly measured through comprehensive physiological testing. Therefore, these findings should not be interpreted as direct evidence that cervical SCS improves airway protection. The strongest and most consistent signal was observed in cough strength, with higher SCSS values during active stimulation in all six patients. In the four conscious patients, FOIS and WST showed directionally concordant score changes during active stimulation, while cSWAL-QoL changed postoperatively. These observations provide a rationale for future prospective investigation to explore the biological plausibility of a neuromodulatory effect on airway protection, but do not establish efficacy or a defined therapeutic effect in this study.

### Integration with previous findings

Previous studies have primarily focused on SCS-mediated restoration of cough through activation of expiratory musculature and enhancement of airway clearance (1–5). Our findings are consistent with this literature and raise the possibility that cervical SCS may be associated with broader clinical proxy measures related to airway protection, including oral intake and swallowing-safety scores. Although prior cough-restoration studies have largely used lower thoracic stimulation to activate expiratory muscles (6, 26–32), cervical stimulation may have a different anatomical relationship to respiratory and airway-protective circuits. The rationale for considering spinal-brainstem network interactions is that cough, swallowing, and respiratory timing are coordinated by medullary centers and are influenced by afferent and efferent pathways passing through the cervical spinal cord. The nucleus tractus solitarius integrates sensory inputs relevant to airway defense, while the nucleus ambiguus contributes to pharyngeal and laryngeal motor control; cervical respiratory circuits and ascending spinal pathways may therefore indirectly influence these brainstem networks. In this context, cervical SCS could theoretically alter the excitability or timing of airway-protective responses through spinal afferent, propriospinal, or respiratory motor pathways. This interpretation remains speculative in the present study because no direct neurophysiological or brainstem recordings were available.

### Exploratory stimulation-state observations

A notable observation in this cohort was a state-associated pattern in SCSS, FOIS, and WST scores. The most favorable scores were generally observed during active stimulation, while OFF-state scores often returned toward baseline. This pattern is compatible with an acute neuromodulatory effect of cervical SCS on airway protective function (17, 18, 33). However, the OFF condition was not based on a standardized washout protocol and was performed only when clinically permissible. Therefore, these findings should be interpreted as exploratory within-subject observations rather than evidence of stimulation-dependent efficacy. The postoperative change in cSWAL-QoL may reflect cumulative functional changes, improved airway management, concomitant rehabilitation or airway care, or broader clinical recovery during the treatment period. The relationship between acute stimulation state and longer-term functional change should be interpreted cautiously. In this small retrospective cohort, the OFF condition did not consistently preserve the full ON-state score pattern for all measures. Therefore, these findings are best understood as preliminary observations of a possible state-associated pattern requiring prospective confirmation.

### Potential mechanisms

The mechanisms underlying these observations are likely multifactorial and remain speculative. Brainstem modulation represents one possible hypothesis rather than a demonstrated mechanism. Cervical SCS may influence ascending spinal projections to key integrative centers involved in airway defense, including the nucleus tractus solitarius and nucleus ambiguus. Modulation of these networks could theoretically alter the excitability of central pattern generators responsible for coordinating cough, swallowing, and respiration (15). A second potential mechanism involves respiratory-swallow coordination. Dysphagia after neurological injury is often associated with impaired coupling between breathing and swallowing. By modulating respiratory rhythm, axial motor output, or expiratory control, cervical SCS may theoretically facilitate safer swallow timing and more effective airway clearance (14). Finally, SCS may enhance the responsiveness of airway protective reflexes by lowering activation thresholds within shared neural circuits (32). These proposed mechanisms should be regarded as hypotheses only, because direct physiological, neurophysiological, or brainstem-level confirmation was not available in the present study.

### Clinical implications

The observed score changes across cough strength, swallowing safety, oral intake, and swallowing-related quality of life suggest that airway protection may represent a candidate topic for future investigation in cervical SCS research. This may be relevant for patients with SCI, ALS, and DOC who are at increased risk of aspiration, secretion retention, and pulmonary complications. The inclusion of comatose patients provides an exploratory perspective because airway-protective cough responses could be assessed in patients unable to voluntarily participate. However, because swallowing function could not be formally evaluated in these patients, the DOC data should be interpreted only as observations of airway protective cough or secretion-clearance responses. These observations support further investigation of cervical SCS and clinical proxy measures of airway protection, but they do not establish a therapeutic indication.

### Limitations

Several limitations should be acknowledged. First, the sample size was extremely small (*n* = 6 overall and *n* = 4 for FOIS, WST, and cSWAL-QoL), which substantially limited statistical power, precludes definitive statistical inference, and restricts the generalizability of the findings. Therefore, all *p* values should be interpreted as nominal, descriptive, and hypothesis-generating rather than confirmatory. Second, the cohort was clinically heterogeneous, including SCI, ALS, and DOC, with different pathophysiology, baseline airway risk, ability to participate in swallowing assessments, potential for recovery, likelihood of disease progression, SCS indications, electrode configurations, stimulation parameters, and assessment timepoints. This heterogeneity may have influenced both baseline impairment and response patterns, thereby preventing disease-specific conclusions. Third, the retrospective case-series design was subject to selection bias, measurement bias, and observational bias. Patients were identified from those who had undergone cervical SCS and had available outcome data, and evaluator blinding or prospective standardization was not possible. Fourth, the absence of a sham-stimulation or randomized control condition prevents causal inference. Fifth, the ON and OFF observations were not based on a standardized stimulation-withdrawal or washout protocol and were performed only when clinically permissible. Sixth, swallowing-specific outcomes were not assessed in patients with DOC. FOIS, WST, and cSWAL-QoL require voluntary participation, reliable oral intake assessment, or patient-reported responses, and therefore were available only in the four conscious patients. Consequently, conclusions regarding swallowing performance and swallowing-related quality of life are limited to conscious patients, whereas the two comatose patients contributed primarily to assessment of cough or airway-protective responses. Seventh, cSWAL-QoL data were not available under separate stimulation ON and OFF conditions, limiting state-dependent interpretation of patient-reported outcomes. Finally, airway protection was inferred from clinical proxy measures rather than assessed by comprehensive objective physiological testing. Objective physiological measurements such as cough peak flow, submental or respiratory electromyography, pharyngeal manometry, video fluoroscopic swallowing study, or fiberoptic endoscopic evaluation of swallowing were not systematically available. Future prospective studies should incorporate these measures together with standardized stimulation washout protocols, blinded assessments, and larger disease-specific cohorts.

## Conclusion

This retrospective case series provides preliminary, hypothesis-generating observations that cervical epidural SCS may be associated with exploratory state-associated changes in clinical proxy measures related to cough strength and swallowing-related aspects of airway protection, particularly in cough-strength score changes. Swallowing-related conclusions are limited to the four conscious patients, whereas the two patients with DOC contributed only to cough or airway-protective response observations. The study does not establish efficacy or mechanism, and definitive statistical inference is precluded by the extremely small sample size, heterogeneous diagnoses, retrospective design, non-standardized ON/OFF observations, and lack of systematic physiological assessments. Prospective controlled studies with larger samples, standardized stimulation protocols, and objective physiological measures are required to determine whether airway protection is a reproducible and clinically meaningful target beyond traditional motor restoration.

## Data Availability

The raw data supporting the conclusions of this article will be made available by the authors, without undue reservation.

## References

[1] DiMarco AF Kowalski KE Geertman RT Hromyak DR. Spinal cord stimulation: a new method to produce an effective cough in patients with spinal cord injury. Am J Respir Crit Care Med. (2006) 173:1386–9. doi: 10.1164/rccm.200601-097CR 16543552 PMC2662977

[2] DiMarco AF Kowalski KE Geertman RT Hromyak DR. Lower thoracic spinal cord stimulation to restore cough in patients with spinal cord injury: results of a National Institutes of Health-sponsored clinical trial. Part I: methodology and effectiveness of expiratory muscle activation. Arch Phys Med Rehabil. (2009) 90:717–25. doi: 10.1016/j.apmr.2008.11.013, 19406289 PMC2813808

[3] DiMarco AF Kowalski KE Geertman RT Hromyak DR Frost FS Creasey GH . Lower thoracic spinal cord stimulation to restore cough in patients with spinal cord injury: results of a National Institutes of Health-sponsored clinical trial. Part II: clinical outcomes. Arch Phys Med Rehabil. (2009) 90:726–32. doi: 10.1016/j.apmr.2008.11.014, 19406290 PMC2809374

[4] DiMarco AF Kowalski KE Hromyak DR Geertman RT. Long-term follow-up of spinal cord stimulation to restore cough in subjects with spinal cord injury. J Spinal Cord Med. (2014) 37:380–8. doi: 10.1179/2045772313y.0000000152, 24090524 PMC4116721

[5] Hachmann JT Calvert JS Grahn PJ Drubach DI Lee KH Lavrov IA. Review of epidural spinal cord stimulation for augmenting cough after spinal cord injury. Front Hum Neurosci. (2017) 11:144. doi: 10.3389/fnhum.2017.00144, 28400726 PMC5368218

[6] DiMarco AF Geertman RT Tabbaa K Nemunaitis GA Kowalski KE. Restoration of cough via spinal cord stimulation improves pulmonary function in tetraplegics. J Spinal Cord Med. (2020) 43:579–85. doi: 10.1080/10790268.2019.1699678, 31809251 PMC7534376

[7] DiMarco AF Geertman RT Nemunaitis GA Kowalski KE. Impact of the cough stimulation system on the care burden and life quality of caregivers of tetraplegics. J Spinal Cord Med. (2023) 46:778–88. doi: 10.1080/10790268.2022.2148845, 37017634 PMC10446787

[8] DiMarco AF Geertman RT Nemunaitis GA Kowalski KE. Effects of restoration of cough via spinal cord stimulation on subject quality of life. J Clin Orthop Trauma. (2022) 34:102027. doi: 10.1016/j.jcot.2022.102027 36212771 PMC9535310

[9] DiMarco AF Geertman RT Tabbaa K Polito RR Kowalski KE. Economic consequences of an implanted neuroprosthesis in subjects with spinal cord injury for restoration of an effective cough. Top Spinal Cord Inj Rehabil. (2017) 23:271–8. doi: 10.1310/sci2303-271 29339903 PMC5562035

[10] Hayashi T Fujiwara Y Ariji Y Sakai H Kubota K Kawano O . Mechanism of dysphagia after acute traumatic cervical spinal cord injury. J Neurotrauma. (2020) 37:2315–9. doi: 10.1089/neu.2020.6983, 32486896

[11] McRae J Morgan S Wallace E Miles A. Oropharyngeal dysphagia in acute cervical spinal cord injury: a literature review. Dysphagia. (2023) 38:1025–38. doi: 10.1007/s00455-022-10535-0, 36374337 PMC10326135

[12] Hachmann JT Grahn PJ Calvert JS Drubach DI Lee KH Lavrov IA. Electrical neuromodulation of the respiratory system after spinal cord injury. Mayo Clin Proc. (2017) 92:1401–14. doi: 10.1016/j.mayocp.2017.04.011, 28781176

[13] Galer EL Huang R Madhavan M Wang E Zhou Y Leiter JC . Cervical epidural electrical stimulation increases respiratory activity through somatostatin-expressing neurons in the dorsal cervical spinal cord in rats. J Neurosci. (2023) 43:419–32. doi: 10.1523/jneurosci.1958-21.2022, 36639888 PMC9864577

[14] Huang R Baca SM Worrell JW Liu X Seo Y Leiter JC . Modulation of respiratory output by cervical epidural stimulation in the anesthetized mouse. J Appl Physiol. (1985) 121:1272–81. doi: 10.1152/japplphysiol.00473.2016 27763875

[15] Malone IG Nosacka RL Nash MA Otto KJ Dale EA. Electrical epidural stimulation of the cervical spinal cord: implications for spinal respiratory neuroplasticity after spinal cord injury. J Neurophysiol. (2021) 126:607–26. doi: 10.1152/jn.00625.2020, 34232771 PMC8409953

[16] Kitamura I Frazure M Iceman K Koike T Pitts T. Stochastic electrical stimulation of the thoracic or cervical regions with surface electrodes facilitates swallow in rats. Front Neurol. (2024) 15:1390524. doi: 10.3389/fneur.2024.1390524, 39045426 PMC11263167

[17] Malone IG Kelly MN Nosacka RL Nash MA Yue S Xue W . Closed-loop, cervical, epidural stimulation elicits respiratory neuroplasticity after spinal cord injury in freely behaving rats. eNeuro. (2022) 9:ENEURO.0426-21.2021. doi: 10.1523/eneuro.0426-21.2021, 35058311 PMC8856702

[18] Mickle AR Peñaloza-Aponte JD Coffey R Hall NA Baekey D Dale EA. Closed-loop cervical epidural stimulation partially restores ipsilesional diaphragm EMG after acute C(2) hemisection. Respir Physiol Neurobiol. (2024) 320:104182. doi: 10.1016/j.resp.2023.104182, 37923238 PMC11135909

[19] Chaw E Shem K Castillo K Wong SL Chang J. Dysphagia and associated respiratory considerations in cervical spinal cord injury. Top Spinal Cord Inj Rehabil Fall. (2012) 18:291–9. doi: 10.1310/sci1804-291 PMC3584789 23459678

[20] Pitts T Iceman KE Huff A Musselwhite MN Frazure ML Young KC . Laryngeal and swallow dysregulation following acute cervical spinal cord injury. J Neurophysiol. (2022) 128:405–17. doi: 10.1152/jn.00469.2021, 35830612 PMC9359645

[21] Shin JC Yoo JH Lee YS Goo HR Kim DH. Dysphagia in cervical spinal cord injury. Spinal Cord. (2011) 49:1008–13. doi: 10.1038/sc.2011.34, 21577216

[22] Wolf C Meiners TH. Dysphagia in patients with acute cervical spinal cord injury. Spinal Cord. (2003) 41:347–53. doi: 10.1038/sj.sc.3101440, 12746741

[23] Xu X Zhang Q Xie Y Yang D Gao F Yuan Y . Coordination between respiration and swallowing in patients with dysphagia after cervical spinal cord injury: an observational case-control study. Am J Speech Lang Pathol. (2024) 33:2572–81. doi: 10.1044/2024_ajslp-24-00135 39240818

[24] Vilardell N Rofes L Nascimento WV Muriana D Palomeras E Clavé P. Cough reflex attenuation and swallowing dysfunction in sub-acute post-stroke patients: prevalence, risk factors, and clinical outcome. Neurogastroenterol Motil. (2017) 29:e12910. doi: 10.1111/nmo.12910, 27424849

[25] Lai X Zhu H Du H Wang J Bo L Huo X. Reliability and validity of the Chinese mandarin version of the swallowing quality of life questionnaire. Dysphagia. (2021) 36:670–9. doi: 10.1007/s00455-020-10181-4, 32865626

[26] Kowalski KE Romaniuk JR Kowalski T DiMarco AF. Effects of expiratory muscle activation via high-frequency spinal cord stimulation. J Appl Physiol (1985). (2017) 123:1525–31. doi: 10.1152/japplphysiol.00402.2017 28935824

[27] Kowalski KE Romaniuk JR Brose SW Richmond MA Kowalski T DiMarco AF. High frequency spinal cord stimulation-new method to restore cough. Respir Physiol Neurobiol. (2016) 232:54–6. doi: 10.1016/j.resp.2016.07.001, 27395446 PMC5012955

[28] Kowalski KE Kowalski T DiMarco AF. Safety assessment of epidural wire electrodes for cough production in a chronic pig model of spinal cord injury. J Neurosci Methods. (2016) 268:98–105. doi: 10.1016/j.jneumeth.2016.05.002, 27168496 PMC4903884

[29] Kowalski KE DiMarco AF. Comparison of wire and disc leads to activate the expiratory muscles in dogs. J Spinal Cord Med. (2011) 34:600–8. doi: 10.1179/2045772311y.0000000039, 22330116 PMC3237287

[30] Kitamura I Kaneko M Sugiyama Y Fuse S Hirano S Iceman K . Efficacy of a gaussian-frequency charge-imbalanced biphasic waveform for facilitating swallowing via thoracic epidural spinal cord stimulation. Neurosci Lett. (2026) 877:138577. doi: 10.1016/j.neulet.2026.138577 41819250

[31] DiMarco AF Kowalski KE Supinski G Romaniuk JR. Mechanism of expiratory muscle activation during lower thoracic spinal cord stimulation. J Appl Physiol (1985). (1985) 92:2341–6. doi: 10.1152/japplphysiol.01231.2001, 12015345

[32] DiMarco AF Kowalski KE. High-frequency spinal cord stimulation in a subacute animal model of spinal cord injury. J Appl Physiol (1985). (2019) 127:98–102. doi: 10.1152/japplphysiol.00006.2019, 31095462 PMC6692743

[33] DiMarco AF Geertman RT Kim C Nemunaitis GA Kowalski KE. Effects of withdrawal and re-application of spinal cord stimulation to restore cough. J Spinal Cord Med. (2025) 48:739–42. doi: 10.1080/10790268.2024.2395082, 39495070 PMC12180311

